# Ran-dependent TPX2 activation promotes acentrosomal microtubule nucleation in neurons

**DOI:** 10.1038/srep42297

**Published:** 2017-02-13

**Authors:** Wen-Shin Chen, Yi-Ju Chen, Yung-An Huang, Bing-Yuan Hsieh, Ho-Chieh Chiu, Pei-Ying Kao, Chih-Yuan Chao, Eric Hwang

**Affiliations:** 1 Department of Biological Science and Technology, National Chiao Tung University, Hsinchu, Taiwan; 2 Institute of Bioinformatics and Systems Biology, National Chiao Tung University, Hsinchu, Taiwan; 3 Center for Bioinformatics Research, National Chiao Tung University, Hsinchu, Taiwan; 4 Institute of Molecular Medicine and Bioengineering, National Chiao Tung University, Hsinchu, Taiwan

## Abstract

The microtubule (MT) cytoskeleton is essential for the formation of morphologically appropriate neurons. The existence of the acentrosomal MT organizing center in neurons has been proposed but its identity remained elusive. Here we provide evidence showing that TPX2 is an important component of this acentrosomal MT organizing center. First, neurite elongation is compromised in TPX2-depleted neurons. In addition, TPX2 localizes to the centrosome and along the neurite shaft bound to MTs. Depleting TPX2 decreases MT formation frequency specifically at the tip and the base of the neurite, and these correlate precisely with the regions where active GTP-bound Ran proteins are enriched. Furthermore, overexpressing the downstream effector of Ran, importin, compromises MT formation and neuronal morphogenesis. Finally, applying a Ran-importin signaling interfering compound phenocopies the effect of TPX2 depletion on MT dynamics. Together, these data suggest a model in which Ran-dependent TPX2 activation promotes acentrosomal MT nucleation in neurons.

During neuronal morphogenesis, post-mitotic neurons transform from their symmetrical shapes into highly polarized ones. These polarized neurons contain long cellular protrusions called neurites that will later develop into axons or dendrites. A functional nervous system depends on the intricate connections between neurites originated from different neurons. Neuronal morphogenesis, like other cellular events in which dynamic cellular asymmetries must be established and maintained, depends on the organization of multiple cytoskeleton systems^1^,^2^,^3^,^4^,^5^. In particular, microtubule (MT) cytoskeleton and its associated proteins play crucial roles during this process^6^,^7^. One of the open questions is the location at which MTs are nucleated in the neuron. Earlier studies indicated that MTs in neurons are nucleated from the centrosome, released by MT severing proteins, and moved into the neurites^8^. More recent data showed that acentrosomal MT nucleation exists in neurons. It has been reported that hardly any MT emanated from the centrosome in mature hippocampal neurons and axon elongation continued even after the centrosome was ablated during early neuronal development^9^. Additionally, Golgi outposts have been demonstrated to nucleate MTs in the dendritic arbor in *Drosophila* da neurons^10^. A recent discovery shows that augmin complex interacts with γ-tubulin ring complex in axons and depleting specific augmin complex subunits reduces MT nucleation in the axon compartment^11^. These data indicate that acentrosomal MT nucleation sites exist in post-mitotic neurons but the precise components and functional location remain unknown.

Ran is a member of the Ras superfamily GTPase that plays fundamental roles in the regulation of transport through the nuclear pore. Ran functions as a molecular switch in which the conversion between GTP-bound and GDP-bound conformations changes how it interacts with its effectors^12^,^13^. GTP-bound Ran (RanGTP) interacts with its effectors and is known as the active form, while the GDP-bound Ran (RanGDP) exhibits low affinity towards the effectors and is known as the inactive form. Besides regulating nucleocytoplasmic transport, it has been well documented that Ran coordinates mitotic spindle assembly^14^,^15^,^16^,^17^,^18^. The effects of Ran on mitotic spindle assembly are mediated through the importin-α/β heterodimer, which binds to the nuclear localization sequence (NLS) on Ran-regulated spindle assembly factors^19^. This interaction inhibits the activity of these spindle assembly factors until the complex is dissociated via the interaction of RanGTP with importin-β^20^,^21^,^22^. Several Ran-regulated spindle assembly factors have been identified and one of the crucial proteins is TPX2. TPX2 is a MT-associated protein known to promote MT nucleation from chromosomes, centrosomes, as well as existing MTs^23^,^24^,^25^. It localizes within the nucleus during interphase and to the centrosomes and spindle MTs during mitosis^26^.

While the effect of Ran on spindle assembly in mitotic cells has been extensively studies, its effect on MT cytoskeleton in post-mitotic neurons has only been scarcely examined. A genome-wide RNAi screen in primary *Drosophila* neurons identified Ran as an important regulator of neuronal morphology^27^. Ran-depleted *Drosophila* neurons displayed excessive neurite branching, neurite blebbing, and reduced neurite outgrowth. Two independent studies showed that Ran-binding protein RanBP9 (or RanBPM) regulated neurite outgrowth. RanBP9 was identified in these studies as the binding partner for the neural cell adhesion molecule L1 and the axon guidance receptor plexin A1. Overexpression of RanBP9 impairs neurite outgrowth in cerebellar neurons and dorsal root ganglion neurons^28^,^29^,^30^. These results suggest that Ran might also be involved in neuronal morphogenesis. It is important to note that high level of RanGTP can be detected in the axoplasm of the sciatic nerve^31^, suggesting that the function of Ran is not restricted to the nucleus in neurons. However, the precise cellular localization of RanGTP in the cytoplasm of neurons has yet to be determined. Interestingly, the Ran-target protein TPX2 has been shown to express in post-mitotic neurons^32^. It localizes to the centrosome in dorsal root ganglion neurons and regulates the MT nucleation from the centrosome via the aPKC-Aurora A-NDEL1 signaling pathway^33^. However, whether TPX2 can be regulated by Ran in neurons remains to be determined.

In this study, we attempted to understand the cellular mechanism of TPX2 on neuronal morphogenesis. We discovered that depleting TPX2 in dissociated neurons caused the reduction in neurite length. In addition to its primary localization to the centrosome, low levels of TPX2 were observed in the entire neuronal cytoplasm bound to the MT cytoskeleton. We analyzed the dynamics of a MT plus-end binding protein EB3 in TPX2-depleted neurons to understand the effect of TPX2 on the MT cytoskeleton. Consistent with the observation in DRG neurons^33^, TPX2 depletion reduced EB3 emanation frequency but did not affect EB3 speed. Furthermore, the decrease of EB3 emanation frequency was only observed at the tip and the base of the neurite. Interestingly, RanGTP was also enriched at the tip of the neurite and in the soma. We also observed that overexpressing importin-α reduced MT nucleation at the tip of the neurite and compromised neuronal morphogenesis. When importazole, a Ran signaling interfering compound, was applied to the neurons, a decrease of MT emanation frequency was detected at the tip and the base of the neurite within minutes. This rapid effect eliminates the possibility of an influence from interfering the nucleocytoplasmic transport. Our data therefore suggest that Ran-dependent TPX2 activation can enhance MT nucleation both at the neurite tip and within soma to promote neuronal morphogenesis.

## Results

### Depleting TPX2 compromises neurite elongation

**Figure 1:**
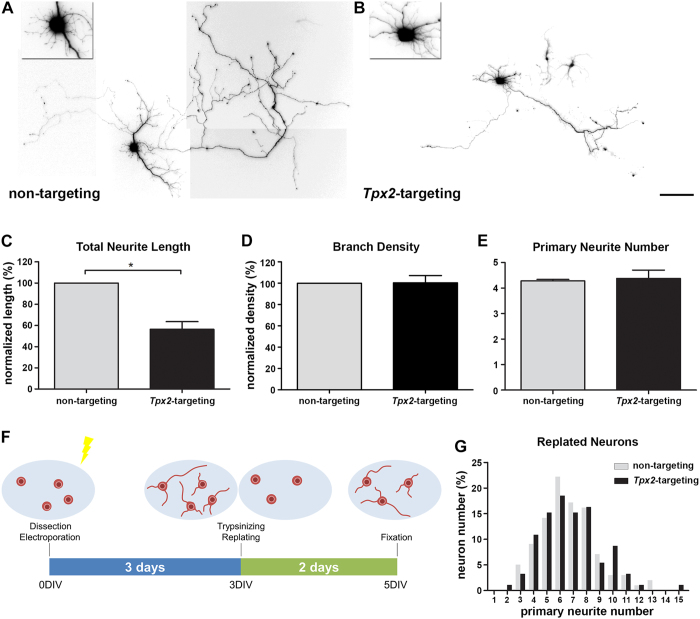
TPX2 depletion affects neurite elongation. Dissociated hippocampal neurons were transfected with plasmid expressing non-targeting control shRNA (**A**) or *Tpx2*-targeting shRNA (**B**) at 2DIV and fixed at 5DIV. The insert shows a magnified image of the soma. Images were inverted and γ-corrected (γ = 0.05, in insert γ = 0.1) to enhance visualization. The scale bar presents 100 μm. Quantification of total neurite length per neuron (**C**), branch number per 200 μm neurite (**D**), and primary neurite number (**E**) of control and TPX2-depleted neurons. *p < 0.05 by two-tailed Student’s *t*-test. Error bars represent SEM from 3 independent experiments. More than 70 neurons were analyzed for each group from 3 independent experiments. (**F**) Schematic diagram of the neuron replating protocol. (**G**) Histogram of primary neurite number distribution of replated neurons. Neuron number of each group was normalized to total neuron number counted (non-targeting = 99, *Tpx2*-targeting = 92).

It has previously been shown that TPX2 depletion resulted in the reduction of neurite length in cultured PNS neurons^33^. To determine if the effect of TPX2 on neurite elongation is also present in CNS neurons, we examined the phenotype of shRNA-mediated TPX2 knockdown in CNS hippocampal neurons. TPX2 depletion was confirmed using both immunofluorescence staining and immunoblotting (Figure S1). Consistent with the previous result, TPX2 knockdown resulted in a reduction in neurite length (Fig. 1A–C). A similar phenotype was observed in embryonal carcinoma cells differentiated neurons using two different shRNA sequences (Figure S2). Additionally, both axon and dendrite lengths were reduced in TPX2 depleted neurons (Figure S3). We did not observe any change in overall neurite branching density (Fig. 1D) or primary neurite number (Fig. 1E) upon TPX2 depletion, suggesting that TPX2 does not influence neurite branching density or initiation. Because neurite initiation occurs soon after neurons attach to the culture surface, our protocol might not allow us to deplete TPX2 in time to interfere with this early morphogenetic event. We therefore developed a method to enable sufficient TPX2 depletion before examining neurite initiation (Fig. 1F). Neurons were transfected immediately after dissociation and cultured for 3 days to allow sufficient TPX2 knockdown. Neurons were subsequently detached from culture surface by trypsin, replated, and cultured for 2 additional days before examination. Even after such a prolonged period of depletion, TPX2 knockdown did not affect neurite initiation (Fig. 1G). These data show that TPX2 is involved in regulating neurite elongation, but not in regulating overall neurite branching density or neurite initiation.

### TPX2 localizes to the centrosome and binds to microtubules along the neurite shaft in neurons

**Figure 2:**
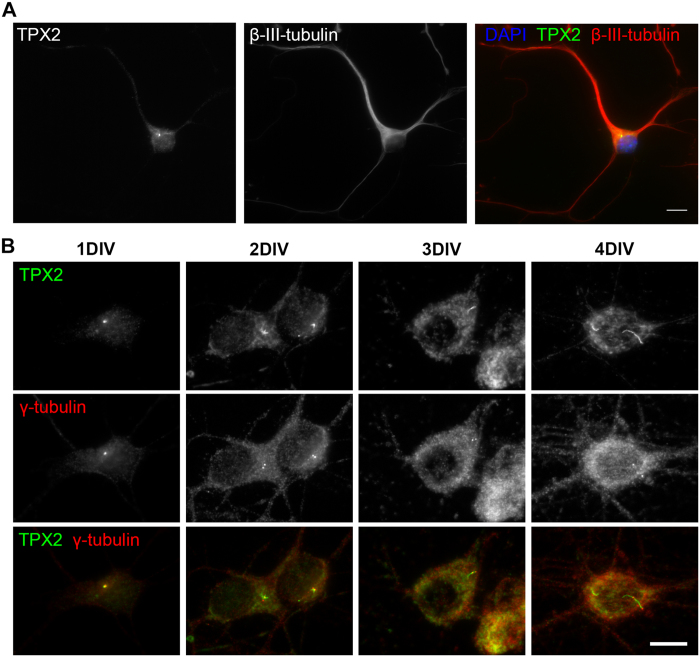
TPX2 localization in dissociated hippocampal neurons. (**A**) 5DIV hippocampal neurons were fixed and stained with anti-TPX2 (left), β-III-tubulin antibody (middle), and DAPI. (**B**) 1~4DIV hippocampal neurons in dissociated culture stained with anti-TPX2 (top row) and anti-γ-tubulin (center row) antibodies. All scale bars represent 10 μm.

To understand how TPX2 regulates neurite elongation, we next examined its localization in cultured neurons. TPX2 has been shown to localize to a variety of locations in post-mitotic neurons, including at the centrosome in cultured dorsal root ganglion neurons^33^ as well as along dendrites and axons in superior cervical ganglion neurons^32^. To determine the localization of TPX2 in neurons, we used immunofluorescence staining to detect endogenous TPX2 in dissociated hippocampal neurons. This antibody was generated using the full length human TPX2 protein^24^, which shares high sequence similarity with the mouse protein. Endogenous TPX2 localized predominately to a single punctum in the soma, with low level of TPX2 observed in the cytosol throughout the neuron (Fig. 2A). The single punctum of TPX2 in the soma colocalized with γ-tubulin, suggesting that TPX2 localizes to the centrosome (Fig. 2B). This is consistent with the previous observation in dorsal root ganglion neurons^33^. Surprisingly, this localization changed over time (Fig. 2B). As neurons matured in culture, the localization of TPX2 changed from a single punctum to an aster-like structure and to an elongated fiber. Despite the change in its localization, TPX2 was observed to connect the γ-tubulin puncta.

**Figure 3:**
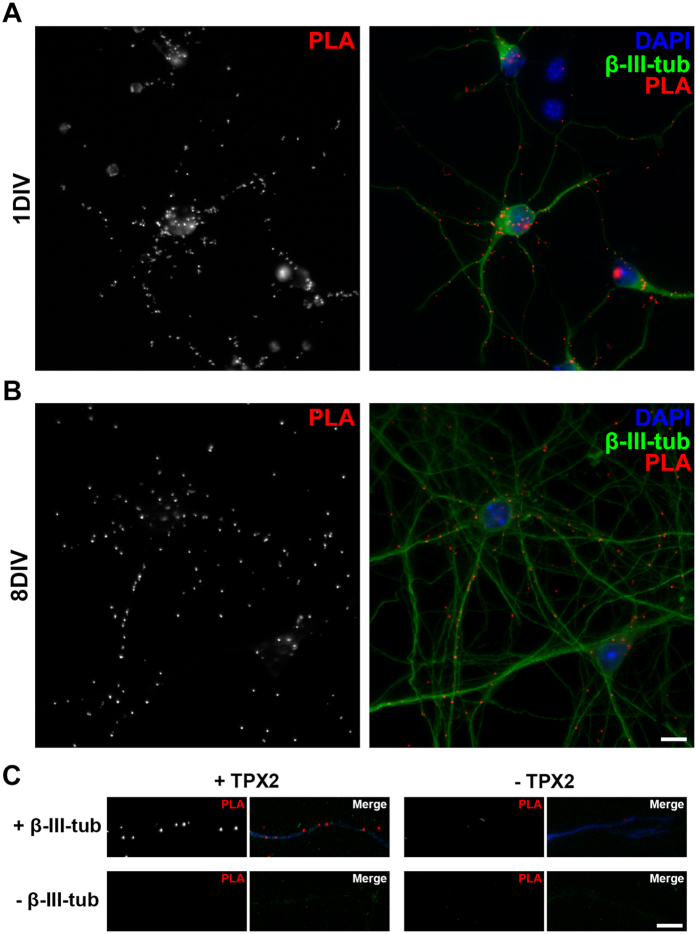
TPX2 localizes to the microtubules along the neurite shaft. Representative images of PLA for TPX2 and β-III-tubulin in 1DIV (**A**) or 8DIV (**B**) dissociated hippocampal neurons. Dylight 488-conjugated secondary antibody was applied after PLA to stain β-III-tubulin antibody and to detect neurons (shown in green). DAPI stain was used to detect the nucleus (shown in blue). (**C**) PLA puncta were produced along the neurite shaft only when anti-TPX2 and anti-β-III-tubulin antibodies were both present. Alexa 405-conjugated secondary antibody was applied after PLA to stain β-III-tubulin antibody and to facilitate neurite visualization (shown in blue). All scale bars represent 10 μm.

The immunofluorescence staining suggests that a minor pool of TPX2 localized along neurites. To confirm and better visualize this TPX2 localization, we conducted *in situ* proximity ligation assay (PLA) in dissociated hippocampal neurons. Because TPX2 is a known MT-associated protein, we reasoned that TPX2 localized along neurites would be bound to the MT cytoskeleton. Antibodies against TPX2 and neuron-specific β-III-tubulin were used in this PLA. If the antibody that recognizes the endogenous TPX2 and the antibody that recognizes β-III-tubulin are in close proximity (<40 nm), a local enzymatic reaction can facilitate the generation of a fluorescent punctum^34^. We first conducted PLA in mitotic cells to confirm the specificity of this assay in detecting TPX2 and α-tubulin interaction. As expected, because TPX2 only localizes to MTs during mitosis, PLA fluorescent puncta were only detectable in cells that entered mitosis (Figure S4). When PLA was performed in dissociated neurons, fluorescent puncta were detected in the soma and along all neurites (Fig. 3A,B), supporting the observation in superior cervical ganglion neurons^32^. PLA fluorescent puncta along neurites could only be observed when both antibodies were present (Fig. 3C), demonstrating the high specificity of our PLA. In addition, PLA between TPX2 and another abundant cytoskeleton protein along the neurite, actin, produced no fluorescent puncta (Figure S5). This shows that TPX2 specifically binds to the MT cytoskeleton along neurites. Both immature (1DIV) and more developed (8DIV) neurons showed MT-bound TPX2 along the neurite shaft. We did not observe significant difference in the distribution of PLA fluorescent puncta along neurites between 1DIV and 8DIV neurons. These results demonstrate that in addition to its primary localization to the centrosome, TPX2 also localizes along the neurite shaft and is bound to the MT cytoskeleton in post-mitotic neurons.

### TPX2 depletion results in the reduction of microtubule emanation frequency at the tip and base of the neurite

**Figure 4:**
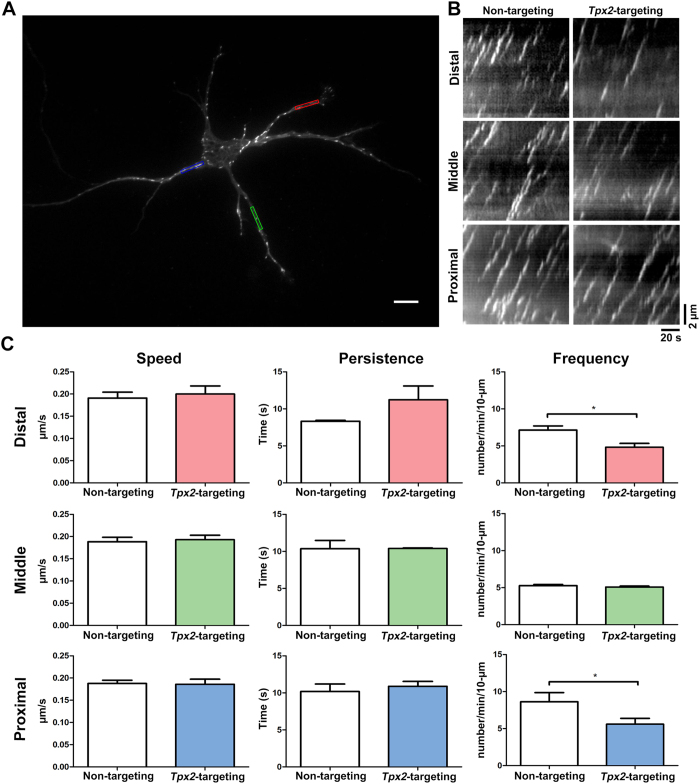
TPX2 depletion reduces microtubule emanating frequency at the tip and base of the neurite. Cortical neurons were cotransfected with plasmids expressing EB3-mCherry and non-targeting or *Tpx2*-targeting shRNA before plating, incubated for 4 days before subjected to live cell imaging. (**A**) The image of a 4DIV cortical neuron expressing EB3-mCherry. The color windows indicated the region from which kymographs were generated. The scale bar represents 10 μm. (**B**) Kymographs of EB3-mCherry in indicated regions of the neurite. (**C**) Quantification of EB3-mCherry dynamics in control and TPX2-depleted neurons. *p < 0.05, two-tailed Student’s *t*-test. Error bars represent SEM from 3 independent repeats. At least 10 neurons were analyzed per condition per repeat.

It has been shown that TPX2 depletion causes the reorganization of MTs in the apical process of G2-phase neural progenitor cells^35^. This, combined with the observation that TPX2 stimulates MT nucleation from the side of existing MTs^23^, led us to hypothesize that MT-bound TPX2 along the neurite shaft can promote MT nucleation inside the neurite. To test this possibility, we introduced mCherry-tagged EB3, a MT plus end-tracking protein, into dissociated cortical neurons to examine MT dynamics. The same approach has been utilized to analyze MT nucleation in both PNS and CNS neurons^11^,^33^. Similar to the previous observation^36^, live cell imaging revealed a characteristic comet-like movement of EB3-mCherry in cortical neurons (Fig. 4A). This indicated that the expression level of EB3-mCherry is modest in our system. No significant alteration in EB3 speed or persistence (defined as the duration of the EB3-mCherry movement before it disappears) was detected when TPX2 was depleted (Fig. 4B and C, Videos 1 and 2); this is consistent with the previous observation in dorsal root ganglion neurons^33^. On the other hand, EB3 emanation frequency was significantly reduced upon TPX2 depletion (Fig. 4B and C, Videos 1 and 2). Surprisingly, the reduction of EB3 emanation frequency was only detected at the tip and the base of the neurite, but not at the middle of the neurite (Fig. 4C). This result indicates that TPX2 promotes MT emanation inside the neurite, but it only functions at specific regions of the neurite.

### GTP-bound Ran concentrates in the neurite tip and the soma

**Figure 5:**
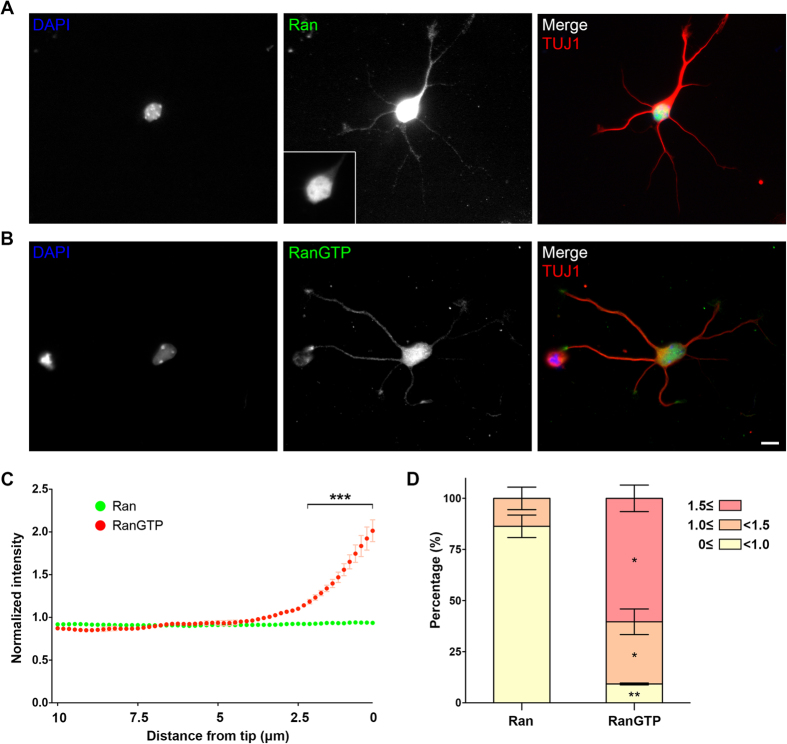
RanGTP is enriched in the tip of the neurite and the soma. (**A**,**B**) Images of 3DIV hippocampal neurons immunofluorescence stained with antibody against Ran (**A**) and RanGTP (**B**), respectively. The inset in middle panel A shows the same image around the soma region with reduced image contrast. All images have the same scale and the scale bar represents 10 μm. (**C**) Ran (green) or RanGTP (red) intensity linescan along a 10 μm stretch from the neurite tip in 3DIV hippocampal neurons. RanGTP accumulated significantly more compared to Ran in the neurite tip. Mean ± SEM from 3 independent experiments, ***p < 0.001, two-way ANOVA followed by Bonferroni post-test. (**D**) To obtain the intensity change at the neurite tip, Ran or RanGTP intensity average within 1 μm from the neurite tip was divided by the intensity along the entire neurite. A ratio above 1.0 indicates an increase, while a ratio below 1.0 indicates a decrease of Ran at the neurite tip. The intensity ratios were classified into three groups. The group with ratio equal to or greater than 1.5 is shown in red, the group with ratio in between 1.5 and 1.0 is shown in orange, and the group with ratio in between 1.0 and 0 is shown in yellow. *p < 0.05, **p < 0.01, two-tailed Student’s *t*-test. Error bars represent SEM from three independent repeats. A total of 131 and 120 neurites were analyzed for Ran and RanGTP from three independent repeats, respectively.

The reduction of MT emanation frequency at the tip and the base of the neurite when TPX2 was depleted suggests that TPX2 is active in these specific regions. One of the TPX2 activators is Ran and its presence inside the neurite has been documented^31^. Since only the GTP-bound Ran (RanGTP) is able to activate TPX2, we used a RanGTP-specific antibody (AR-12)^37^ to determine the localization of RanGTP in neurons. This antibody has previously been used to detect the localization of RanGTP in mitotic cells^38^,^39^. An antibody that recognizes both RanGTP and RanGDP was used to localize both forms of Ran. The majority of Ran localized within the nucleus (Fig. 5A), with low level detectable along the neurite. On the other hand, RanGTP localized in the soma and to the tip of the neurites (Fig. 5B). To quantify this localization, linescans along all neurites were performed; the intensity of RanGTP showed a marked increase at the tip of neurites (Fig. 5C). On the contrary, the intensity of Ran remained constant along the neurite; this indicated that the increase of RanGTP at the neurite tip was not simply due to the increase in the cytoplasmic volume. We then compared Ran and RanGTP signal intensity 1 μm from neurite tips to the average intensity along the entire neurite shaft (Fig. 5D). Sixty percent of the neurites exhibited >1.5-fold increase in the RanGTP signal at the neurite tip. Approximately 30% of the neurites exhibited a slight increase (between 1- and 1.5-fold), while less than 10% of the neurites showed lower RanGTP intensity at the tip. For the Ran signal, most neurites (>80%) exhibited lower intensity at the neurite tip. None of the neurites exhibited higher than 1.5-fold increase of Ran at the neurite tip. These results reveal that RanGTP is concentrated in the soma and at the tip of the neurite.

### Importin regulates microtubule emanation and neuronal morphogenesis

**Figure 6:**
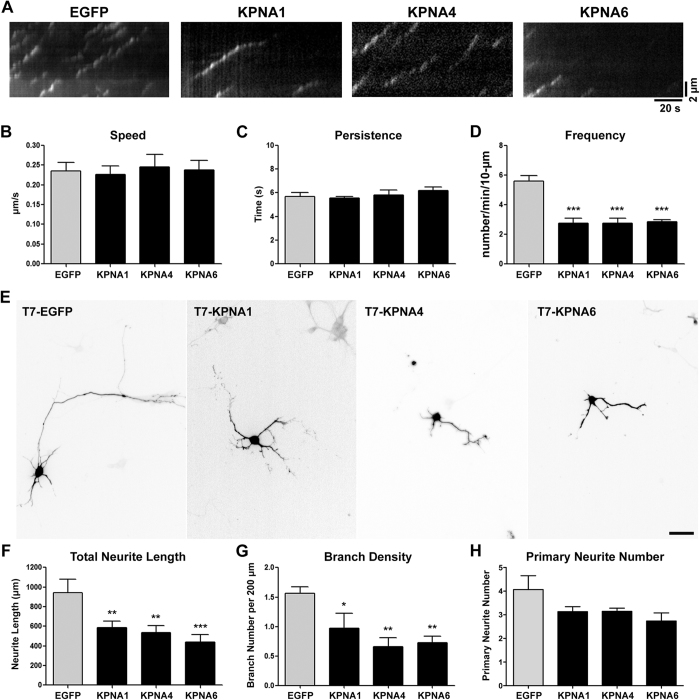
Overexpressing importin-α reduces microtubule emanating frequency and compromises neuronal morphogenesis. Cortical neurons were cotransfected with plasmids overexpressing importin-α (KPNA1, 4, 6) and EB3-mCherry before plating, incubated for 2 days before subjected to live cell imaging. (**A**) Kymographs of EB3-mCherry at the tip of neurites. (**B**–**D**) Quantification of EB3-mCherry dynamics in EGFP (control) and importin-α-overexpressing neurons. At least 10 neurons were analyzed per condition per repeat. (**E**) Representative images of dissociated hippocampal neurons transfected with plasmid overexpressing KPNA1, KPNA4, or KPNA6 at 2DIV and fixed at 4DIV. Images were inverted to enhance visualization. The scale bar presents 50 μm. Quantification of total neurite length (**F**), branch number per 200 μm neurite (**G**), and primary neurite number (**H**) of EGFP- and importin-α-overexpressing neurons. More than 50 neurons were analyzed for each group from 3 independent experiments. *p < 0.05, **p < 0.01, ***p < 0.001, one-way ANOVA followed by Dunnett’s comparison to the control group. Error bars represent SEM from 3 independent experiments.

The reduction of MT emanation frequency at specific regions of the neurite and the distribution of RanGTP at the similar location suggest that Ran GTPase may be required to activate otherwise inhibited TPX2. It has been documented that importin-α/β heterodimer binds to TPX2 and inhibits its MT nucleation activity^40^. To determine if TPX2 is indeed inhibited by importins in neurons, we first examined whether importin-α and importin-β are present in the cytoplasm of the neuron. Consistent with the previous report^41^, both importin-α and β can be observed in the cytoplasm and along the neurite in hippocampal neurons (Figure S6). In addition, we confirmed the presence of the Ran-importin-β complex in the cytoplasm of the neuron using PLA with anti-Ran (which recognizes both RanGTP and RanGDP) and anti-importin-β antibodies (Figure S7). Interestingly, these PLA puncta were often detected in the soma and at the neurite tips, supporting the presence of RanGTP at these regions. Our results thus far support the hypothesis that RanGTP binds to importin-β and releases TPX2 from the inhibitory importin heterodimers. If this hypothesis is true, an excessive amount of importin-α would interfere with TPX2 activation and cause a reduction in MT nucleation at the neurite tip. We chose to overexpress KPNA1, 4, 6 in dissociated neurons because they are the most abundant importin-α isoforms in the central nervous system^42^. Consistent with our hypothesis, overexpressing KPNA1, 4, 6 reduced EB3 emanation frequency at the tip of the neurite (Fig. 6A–D). This suggests that importin-α is involved in regulating TPX2 activity inside the neurite. In addition, KPNA1, 4, 6 overexpression reduced neurite length and neurite branching density (Fig. 6E–H), showing that importin-α influences neuronal morphogenesis. Consistent with the effect of TPX2 depletion, importin-α overexpression affected both axon and dendrite length (Figure S8). Interestingly, the overexpression of KPNA1, 4, 6 affects the neurite branching density but TPX2 depletion does not. This suggests that importin-α inhibits other target proteins in addition to TPX2.

### Interfering with the interaction between RanGTP and importin-β reduces microtubule emanation frequency at the tip and base of the neurites

**Figure 7:**
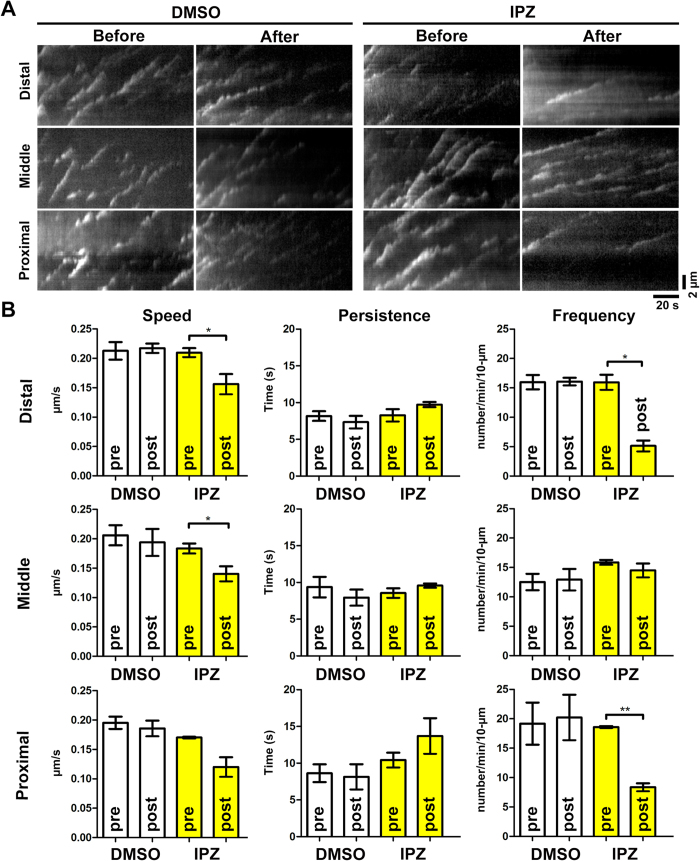
Importazole reduces microtubule emanating frequency at the tip and the base of the neurite. Cortical neurons were transfected with plasmid expressing EB3-mCherry before plating, incubated for 4 days before subjected to live cell imaging in the presence of 15 μM importazole (IPZ). (**A**) Kymographs of EB3-mCherry in indicated regions of the neurite. (**B**) Quantification of EB3-mCherry dynamics in control (DMSO) or 15 μM IPZ (IPZ) treated neurons. EB3 dynamic properties before IPZ application (pre) and those after IPZ application (post) are compared. *p < 0.05, **p < 0.01, two-tailed Student’s *t*-test. Error bars represent SEM from 3 independent repeats. 6 neurons were analyzed per condition per repeat.

To demonstrate that Ran does regulate the activity of TPX2 inside the neurite, we need to rapidly block Ran-mediated signaling before its effect on nucleocytoplasmic transport takes place. Importazole, a cell-permeable blocker that can rapidly interfere the interaction between RanGTP and importin-β^43^, provided an ideal reagent for our analysis. Importazole has been shown to block spindle assembly in *Xenopus* egg extract and impair mitotic importin cargo release in HeLa cells, indicating its efficacy in blocking the activation of Ran-mediated spindle assembly factors such as TPX2. If RanGTP activated TPX2 by releasing it from the inhibitory importin-α/β heterodimer, applying importazole would prevent this release and reduce MT nucleation at the tip and base of the neurite. When importazole was applied to the dissociated neurons, we observed a concentration-dependent neurite retraction effect (Figure S9). Given the effect of importazole on nucleocytoplasmic transport, this result did not come as a surprise. To examine the effect of interfering with RanGTP-mediated TPX2 activation and MT nucleation, we selected the importazole concentration (15 μM) at which neurite retraction was not detectable even after 2 hours of incubation. We again used EB3-mCherry to analyze the MT dynamics in cortical neurons. Within 5 minutes of 15 μM importazole application, EB3 emanation frequency at the proximal and distal neurite was significantly reduced (Fig. 7, Videos 3 and 4). However, EB3 emanation frequency remained constant at the middle of the neurite. This observation indicated that interfering the interaction of RanGTP and importin-β reduced the TPX2-induced MT nucleation in proximal and distal neurite. Interestingly, we also observed a significant reduction in EB3 speed. This indicates that importazole affects MT polymerization rate in neurons. We do not believe this compromises our conclusion that RanGTP activates TPX2 inside the neurite. If importazole affects MT nucleation in the neurite, it would affect nucleation along the entire neurite, not just the distal and proximal regions. Additionally, we examined the effect of importazole on MT dynamics in interphase cells in which TPX2 is sequestered inside the nucleus. In these importazole-treated interphase cells, EB3 emanation frequency remained constant but EB3 speed was significantly reduced (data not shown). This indicates that the effect of importazole on MT nucleation was separable from its effect on MT polymerization.

To determine whether Ran regulates acentrosomal MT nucleation, we decided to introduce a constitutively active RanQ69L or a dominant negative RanT24N mutant in dissociated hippocampal neurons. The RanQ69L mutation severely reduces the GTPase activity of Ran^44^; hence the protein is always in the active, GTP-bound state. The RanT24N mutation disrupts the protein’s affinity for GTP^44^. As a result, RanT24N binds strongly to RanGEF and blocks endogenous Ran proteins from being activated. We reasoned that if Ran promotes acentrosomal MT nucleation by activating TPX2, the constitutively active RanQ69L would generate a higher density of MTs along the neurite than the dominant negative RanT24N. The β-III-tubulin signal intensity along the primary neurite was used to quantify the MT density in neurons. Consistent with our hypothesis, neurons overexpressing RanQ69L possess a higher intensity of β-III-tubulin signal than those overexpressing RanT24N (Figure S10). These results show that Ran regulates acentrosomal MT formation and interfering with Ran signaling compromises this activity.

## Discussion

**Figure 8:**
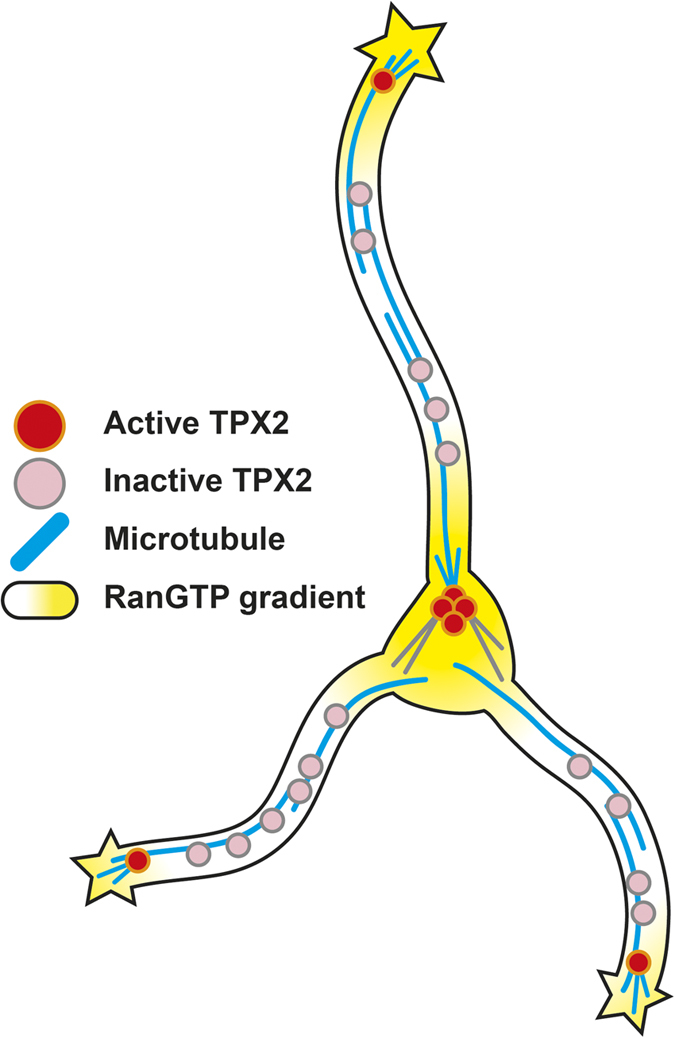
Schematic model for the Ran-activated TPX2 in microtubule nucleation. MT-bound TPX2 molecules are activated by the RanGTP gradient, which is concentrated at the tip of the neurite and in the soma.

In this study, we discovered that TPX2 participated in neuronal morphogenesis and localized to MTs along the neurite shaft. Depleting TPX2 in neurons causes a decrease in MT emanation frequency specifically at the distal and proximal neurite regions. Interestingly, these neurite regions overlap with the distribution of RanGTP in the neuron. Overexpressing importin-α, the downstream target of Ran, decreases MT emanation frequency inside the neurite and compromises neuronal morphogenesis. Treating neurons with a Ran-importin-β interfering compound phenocopies the effect of TPX2 depletion on MT emanation. These data suggest a model in which the spatially restricted RanGTP gradient activates the MT-bound TPX2 and promotes MT nucleation in the soma (potentially from the centrosome) as well as in the distal neurite (Fig. 8).

Using immunofluorescence staining, we were able to detect TPX2 in the soma and along the neurite shaft. Interestingly, while the soma-localized TPX2 initially colocalized with γ-tubulin, its localization changed over time. As neurons developed in culture, the localization of TPX2 changed from a single punctum to an aster-like structure and to an elongated fiber. In addition, TPX2 was observed to connect the γ-tubulin puncta no matter its localization. This peculiar localization is reminiscent of the Golgi apparatus seen in migrating neurons^45^. A recent discovery showed that Golgi matrix protein GM130 interacts with and sequesters importin-α, allowing TPX2 to stimulate local MT nucleation around the Golgi apparatus^46^. This and our observation both demonstrate that local TPX2 activation can promote acentrosomal MT nucleation.

The decreased of MT nucleation frequency was detected at the tip and the base of the neurite when TPX2 was depleted in cortical neurons. This is consistent with the observation that TPX2 depletion reduced MT emanation from the centrosome in dorsal root ganglion neurons^33^. Because atypical protein kinase C (aPKC) has been shown to activate Aurora A kinase at the centrosome in neurons^33^, it will be interesting to determine whether Ran and aPKC work together or independently at the centrosome to promote MT nucleation. One possible way of testing this is to utilize an N-terminal truncated form of TPX2 which lacks the Aurora A-binding domain but contains the NLS. This truncated TPX2 still associates with and nucleates MTs^23^,^47^, and it remains under the regulation of Ran but not Aurora A. One can deplete the endogenous TPX2 in neurons and perform rescue experiment using this TPX2 truncation. A lack of rescue in EB3 emanation frequency near the centrosome would indicate that Ran and aPKC act together in activating TPX2.

Besides the function of Ran and TPX2 around the centrosome, our observation also indicates the existence of acentrosomal MT nucleation sites within the neurite. This is in support of the report that the centrosome loses its function as a MT organizing center while the acentrosomal MT nucleation sites take over this role during neuronal development^9^. Furthermore, it has recently been shown that γ-tubulin and augmin are involved in acentrosomal MT nucleation in neurons^11^. Given that branching MT nucleation from existing MTs requires γ-tubulin and augmin, and is stimulated by RanGTP and TPX2^23^, it is possible that acentrosomal MT nucleation in post-mitotic neurons utilizes very similar mechanism as in the mitotic spindle.

It will be important to examine whether the effect of RanGTP on acentrosomal MT nucleation can influence specific morphological features of neurons (e.g. neurite initiation or branching). However, due to Ran’s role in nucleocytoplasmic transport, the traditional approach of using the constitutively active or the dominant negative Ran mutants will likely fail. One potential method to achieve such goal is to utilize an optogenetic tool to spatially control the activation of Ran. Fusing the LOV2 domain of a phototrophin to the constitutively active Rac1 has previously been demonstrated to allow photoactivation of Rac1 inside the cell^48^. Given the overall structural similarity between Rac1 and Ran, one might be able to construct a photoactivatable Ran using a similar strategy. With such a optogenetic tool at hand, one would be able to activate Ran at specific location along the neurite and ask whether the local elevation of active Ran can influence neuronal morphogenesis.

While the presence of RanGTP along the axon has been documented^31^, the exact distribution of RanGTP along the neurite was not known. Our study shows that the soma and neurite tips have the highest concentration of RanGTP. This result suggests the presence of a cytoplasmic RanGEF at the tip of the neurite. RanBP10, a cytoplasmic RanGEF that associates with megakaryocyte MT^49^, has been proposed to be a likely candidate as the cytoplasmic RanGEF in neurons^50^. Additionally, RanBP10 depletion in megakaryocytes resulted in the disruption of the MT cytoskeleton. This suggests that spatially restricted RanGTP generation by RanBP10 can influence MT organization and dynamics. Therefore, it will be of interest to examine RanBP10 localization and the effect of RanBP10 depletion on RanGTP distribution in neurons.

It is interesting to point out that RanGAP has been reported to concentrate to the tip of the growing axon in dissociated dorsal root ganglion neurons^31^. This indicates that both RanGAP and RanGEF localize to the tip of the neurite. While the colocalization of RanGAP and RanGEF in neurons remains to be examined, it is tempting to speculate that RanGAP and RanGEF exhibit spatially distinct localization in the neurite tip with RanGEF localizes to the growth cone tip (e.g. lamellipodia or filopodia) and RanGAP localizes to the growth cone wrist. This kind of localization would allow neurons to restrict RanGTP to the neurite tip and prevents undesired MT nucleation from occurring elsewhere in the neurite.

Our model predicts the reduction of cytoplasmic RanGTP would diminish the pool of activated TPX2, decrease MT nucleation, and eventually lead to the decrease in neurite length. This is consistent with the morphological phenotype observed in Ran-depleted neurons, which showed a decrease in neurite length^27^. When Ran signaling interfering compound importazole was applied to the neurons, the effect on MT emanation can be observed within minutes. This rapid response supports the presence of the Ran-activated MT nucleation site within the neurite tip and near the soma. Additionally, this result also indicates that TPX2 requires constant Ran-mediated activation to sustain MT nucleation. This kind of regulation mechanism would allow neurons to efficiently utilize MT cytoskeleton for morphogenesis at specific sites. Previous studies showed that the Ran-binding protein RanBP9 regulated neurite outgrowth via interaction with neural cell adhesion molecule L1 and the axon guidance receptor plexin A1. Overexpression of RanBP9 impaired neurite outgrowth in cerebellar neurons and dorsal root ganglion neurons^28^,^29^,^30^. Given that the GEF domains of RanBP9 and RanBP10 are highly conserved and RanBP9 might also harbor RanGEF activity^50^, it might be possible that RanBP9 regulates neuronal morphogenesis not only via its interaction with adhesion molecules but also by regulating RanGTP production.

## Methods

### Antibodies and reagents

TPX2 antibody was a kind gift from Oliver Gruss^24^. Anti-RanGTP antibody (AR-12) was a kind gift from Ian Macara^37^. Mouse anti-α-tubulin monoclonal antibody DM1A (T6199) and Duolink reagents were from Sigma (St Louis, MO). Mouse anti-β-III-tubulin antibody TUJ1 (MMS-435P) and mouse anti-neurofilament monoclonal antibody SMI312 (SMI-312R) were from Covance (Princeton, NJ). Mouse anti-γ-tubulin antibody GTU-88 (GTX11316), HRP-labeled goat-anti-rabbit IgG antibody (GTX213110), and HRP-labeled goat-anti-mouse IgG antibody (GTX213111) were from GeneTex (Irvine, CA). Mouse anti-actin antibody C4 (MAB1501), mouse anti-GAPDH antibody 6C5 (MAB374), and rabbit anti-MAP2 polyclonal antibody (AB5622) were from Millipore (Billerica, MA). Rabbit anti-Ran polyclonal antibody (ab31118) and rabbit anti-importin β1 (ab45938) were from Abcam (Cambridge, UK). Mouse anti-importin-α antibody (sc-55538) was from Santa Cruz Biotechnology (Santa Cruz, CA). Dylight-conjugated secondary antibodies were from Jackson ImmunoResearch (West Grove, PA). Alexa Fluor-conjugated secondary antibodies were from Life Technologies (Carlsbad, CA).

### shRNA and overexpression plasmids construction

The shRNA sequences used in this study are as follow: non-targeting shRNA GCGCGCTATGTAGGATTCGTT (pSUPER) and CGCGATCGTAATCACCCGAGT (pLKO); *Tpx2*-targeting shRNA CAAGGCAAATCCAATACGGAA (pSUPER) and GAGAGCTTGTTACCCTCCAAA (pLKO). shRNAs were inserted into the pSUPER-CAG-EGFP plasmid under the H1 promoter or pLKO.1 plasmid under the U6 promoter. Importin overexpressing plasmids (pCAG-T7-KPNA1, 4, 6) were cloned by inserting T7-KPNA1, 4, 6 from pCMVTNT-T7-KPNA1, 4, 6^51^ into pCAG-AcGFP-C3 vector using XhoI and XmaI.

### P19 cell culture, differentiation, and transfection

Embryonal carcinoma P19 cells were maintained at 37 °C in 5% CO_2_ in MEM supplemented with 1 mM sodium pyruvate, and 10% fetal bovine serum (FBS). The transfection with Lipofectamine 2000 was performed in 96-well plates. Each well on the plate was pre-loaded with 0.64 μg of shRNA-expressing plasmid and 0.36 μg of proneural gene-expressing plasmid in 25 μL serum-free MEM. Then 0.5 μL Lipofectamine 2000 (premixed with 25 μL serum-free MEM) was added to each well to make a 50 μL transfection mixture. After 20 minutes to allow liposome formation, 2 × 10^4^ P19 cells in 200 μL MEM plus 5% FBS were added to each well and maintain in a 37 °C, 5% CO_2_ incubator. Four days post-transfection, P19 cell cultures were fixed with 3.7% formaldehyde in PBS.

### Neuron culture and transfection

All animal experimental procedures were approved by the Institutional Animal Care and Use Committee (IACUC) and in accordance with the Guide for the Care and Use of Laboratory Animals of National Chiao Tung University.

Hippocampal or cortical neuron culture were prepared as described with slight modification^52^. Briefly, brains from E18 mouse pups (C57BL/6) were dissected, digested with trypsin-EDTA, and triturated. Dissociated neurons were seeded onto poly-L-lysine-coated coverslip (3 × 10^4^ neurons per cm^2^). Plasmids were introduced into hippocampal or cortical neurons using Nucleofector II (Lonza, Basel, Switzerland) immediately after dissociation. In some cases, neurons were transfected at 2DIV using Lipofectamine 2000. Transfected cells were incubated for 6 hours and the medium containing transfection mixture was replaced with fresh neurobasal medium. Neurons were fixed at indicated days post-transfection.

For hippocampal neurons replating experiment, Nucleofector transfected hippocampal neurons were seeded in 35 mm dish and incubated for 3 days. Culture medium was replaced with 0.25% trypsin-EDTA. After 5 minutes of incubation, MEM supplemented with 5% FBS, 0.6% D-glucose, and 2 mM L-glutamine was added to neutralize trypsin. Cell suspension was transferred into a 15 mL conical tube and was centrifuged at 80 g for 10 minutes at room temperature. Supernatant was discarded and cells were resuspended in neuronal plating medium. 6 × 10^4^ cells were plated onto a PLL-coated coverslip inside the 24-well plate. Culture medium was replaced with neurobasal medium plus B27 supplement after 2 hours and coverslips were inverted upside down. Neurons were incubated for additional 2 days before fixation.

### Immunoblotting

pSUPER-CAG-EGFP shRNA plasmids were transfected into P19 cells with Lipofectamine 2000. Cell lysate was harvested after 2 days of incubation and subjected to SDS-PAGE. Proteins were then transferred to a PVDF membrane and blocked with 5% dried skimmed milk in TTBS for 30 minutes at room temperature. TPX2 and GAPDH were detected by anti-TPX2 antibody (1:2000) and anti-GAPDH antibody 6C5 (1:500) diluted in TTBS followed by HRP-labeled goat-anti-rabbit IgG (1:2000) or HRP-labeled goat-anti-mouse IgG (1:2000) secondary antibody, respectively. The protein bands were visualized using Western Lightning Plus-ECL, Enhanced Chemiluminescence Substrate (PerkinElmer, Waltham, MA).

### Indirect immunofluorescence staining

Cells on coverslips were fixed with 3.7% formaldehyde for 15 minutes at 37 °C and then washed three times with PBS. Fixed cells were permeabilized with 0.25% triton X-100 in PBS for 5 minutes at room temperature or extracted in −20 °C methanol for 10 minutes. For γ-tubulin staining, cells were fixed with −20 °C methanol for 2 minutes. For experiments that required cytosolic extraction, cells on coverslips were extracted in 0.1% triton X-100 in PIPES buffer (0.1 M PIPES pH6.9, 1 mM MgCl_2_, and 1 mM EGTA) for 15 seconds and then washed with PIPES buffer once. After washing, cells were fixed immediately in 3.7% formaldehyde in PIPES buffer at 37 °C for 30 minutes and then washed with PBS three times. After being washed three times with PBS, cells were blocked with 10% BSA in PBS for 30 minutes at 37 °C. Coverslips with cells were incubated for 1 hour at 37 °C with different primary antibodies: anti-actin (1:500), anti-TPX2 (1:2000), anti-β-III-tubulin antibody (1:4000), anti-γ-tubulin (1:1000), anti-Ran (1:50), anti-RanGTP (1:100), anti-importin-β (1:1000), anti-neurofilament (1:1000), and anti-MAP2 (1:1000). After primary antibody incubation, cells were incubated with fluorophore-conjugated secondary antibodies (1:1000). All antibodies were diluted in 2% BSA in PBS. Coverslips with cells were washed with PBS three times and mounted with Fluoromount onto glass slides.

### *In situ* proximity ligation assay (PLA)

Hippocampal neurons were fixed in −20 °C methanol (for TPX2 and β-III-tubulin) or 3.7% paraformaldehyde (for Ran and importin-β as well as TPX2 and actin) at 1DIV and 8DIV. Neurons were washed with PBS and then blocked in a chamber with Duolink II Blocking Solution for 30 min at 37 °C. Primary antibodies used for different experiments were diluted in PBS containing 2% BSA at aforementioned dilutions and incubated for 1 hour at 37 °C. Cells were incubated with PLA probes diluted in Antibody Diluent for 1 hour at 37 °C. Subsequent procedures were conducted according to the manufacturer’s instructions. Samples were air dried and mounted onto glass slides.

### Microscopy acquisition

Immunofluorescence stained images were acquired with an Olympus IX-71 inverted microscope equipped with a CoolLED fluorescent light source, a Hamamatsu ORCA-R2 camera, and MetaMorph software. 10 × 0.4 N.A., 40 × 0.95 N.A., or 60 × 1.35 N.A. Plan Apochromat objective lenses were used to collect fluorescence images.

Live cell imaging was performed on a Nikon Eclipse-Ti inverted microscope equipping with a TIRF illuminator, a built-in Perfect Focus system, and a Tokai Hit TIZHB live cell chamber. Images were acquired using a 60 × 1.49 N.A. Plan Apochromat objective lens, a 561 nm DPSS laser, a Photometrics CoolSNAP HQ2 camera, and Nikon NIS-Elements imaging software. Images were acquired every 500 milliseconds over a 2-minute period. Only the neurons with clear EB3 comets were imaged.

### Image analysis

For neurite length analysis of P19 neurons, β-III-tubulin stained images were analyzed using ImageJ plug-in NeurphologyJ^53^. For neurite length analysis of hippocampal neurons, neurites were manually traced with the ImageJ plugin NeuronJ 1.4.1^54^. Only neurons expressing both EGFP and β-III-tubulin were analyzed. Neurites were measured from the edge of the soma to the wrist of the growth cone. Only neurites longer than its soma diameter were analyzed.

To analyze Ran signal along neurites, immunofluorescence signals of Ran or RanGTP of 3DIV hippocampal neurons were manually traced and quantified with ImageJ by drawing segment lines in six pixels width along the neurite. Only neurites longer than its soma diameter and not in contact with other neurites were analyzed. The segment lines along neurites were measured from the edge of the soma to the wrist of the growth cone. If a neurite did not have a marked growth cone, the segment line was measured to the tip. Only β-III-tubulin-positive neurons were used for this analysis.

For EB3 dynamics analysis, NIS-Elements software was used to generate the kymograph and to quantify the EB3 motility. A window 10 μm in length and 7 pixels in width was used to generate the kymograph. For proximal neurite analysis, the kymograph window started from the edge of the soma and extended outwards. For mid-neurite analysis, the kymograph window was centered at the midpoint of the neurite. For distal neurite analysis, the kymograph window started at the wrist of the growth cone and extended inwards. The speed and persistence time of EB3-mCherry were quantified from the kymograph by drawing a line along an EB3-mCherry event. Only EB3-mCherry movements that could be followed clearly for equal or more than four frames (1.5 seconds) were defined as an event. The emanating frequency of EB3-mCherry was quantified from the kymograph by counting the number of EB3-mCherry events per minute.

### Statistical analysis

All statistical analyses were performed using GraphPad Prism 5. Significant difference between the means was calculated with two-tailed Student’s *t*-test, one-way ANOVA followed by Dunnett’s post-test, or two-way ANOVA followed by Bonferroni post-test.

## Additional Information

**How to cite this article**: Chen, W.-S. *et al*. Ran-dependent TPX2 activation promotes acentrosomal microtubule nucleation in neurons. *Sci. Rep.* **7**, 42297; doi: 10.1038/srep42297 (2017).

**Publisher's note:** Springer Nature remains neutral with regard to jurisdictional claims in published maps and institutional affiliations.

## Supplementary Material

Supplementary Materials

Supplementary Video 1

Supplementary Video 2

Supplementary Video 3

Supplementary Video 4
