# Supplementary material for: Ran-dependent TPX2 activation promotes acentrosomal microtubule nucleation in neurons
Source: Sci Rep. 2017 Feb 13;7:42297. doi: 10.1038/srep42297 (PMC5304320; doi:10.1038/srep42297)
Supplement: Supplementary Materials [file srep42297-s1.pdf]

Supplementary Materials

Ran-dependent TPX2 activation promotes acentrosomal microtubule nucleation in neurons

Wen-Shin Chen[1,2,3,#], Yi-Ju Chen[4,#], Yung-An Huang[1,2,#], Bing-Yuan Hsieh[4], Ho-Chieh Chiu[4], Pei-Ying Kao[2], Chih-Yuan Chao[4], Eric Hwang[1,2,3,4,*]

[1]Department of Biological Science and Technology, National Chiao Tung University, Hsinchu, Taiwan

[2]Institute of Bioinformatics and Systems Biology, National Chiao Tung University, Hsinchu, Taiwan

[3]Center for Bioinformatics Research, National Chiao Tung University, Hsinchu, Taiwan

[4]Institute of Molecular Medicine and Bioengineering, National Chiao Tung University, Hsinchu, Taiwan

[#]These authors contributed equally to this work

[*]To whom correspondence should be addressed:
hwangeric@mail.nctu.edu.tw

This file includes:
Video Legends for Video 1-4, Supplementary Figures and Legends for S1-S10

Video Legends

Video 1. Live cell imaging of EB3-mCherry in neurons expressing non-targeting shRNA. Mouse cortical neurons were cotransfected with plasmids expressing EB3-mCherry and non-targeting shRNA before plating, incubated for 4 days before subjected to live cell imaging. Images were acquired every 500 milliseconds over a 2-minute period. The scale bar represents 10 μm.
Link: https://figshare.com/s/58403199b8b0d9496973

Video 2. Live cell imaging of EB3-mCherry in neurons expressing *Tpx2*-targeting shRNA showed reduced emanation frequency. Mouse cortical neurons were cotransfected with plasmids expressing EB3-mCherry and *Tpx2*-targeting shRNA before plating, incubated for 4 days before subjected to live cell imaging. Images were acquired every 500 milliseconds over a 2-minute period. The scale bar represents 10 μm.
Link: https://figshare.com/s/c8bbb9dcb36dfc26fb00

Video 3. Live cell imaging of EB3-mCherry in neurons before and after the treatment of DMSO. Mouse cortical neurons were transfected with plasmid expressing EB3-mCherry before plating, incubated for 4 days and subjected to live cell imaging before and after the treatment of DMSO. Images were acquired every 500 milliseconds over a 2-minute period both before and after DMSO application. The scale bar represents 10 μm.
Link: https://figshare.com/s/7c0aa661757749l3873c

Video 4. Live cell imaging of EB3-mCherry in neurons before and after the treatment of importazole. Mouse cortical neurons were transfected with plasmid expressing EB3-mCherry before plating, incubated for 4 days and subjected to live cell imaging before and after the treatment of importazole. Images were acquired every 500 milliseconds over a 2-minute period both before and after importazole application. The scale bar represents 10 μm.
Link: https://figshare.com/s/8fe65e23fa5e1b303487

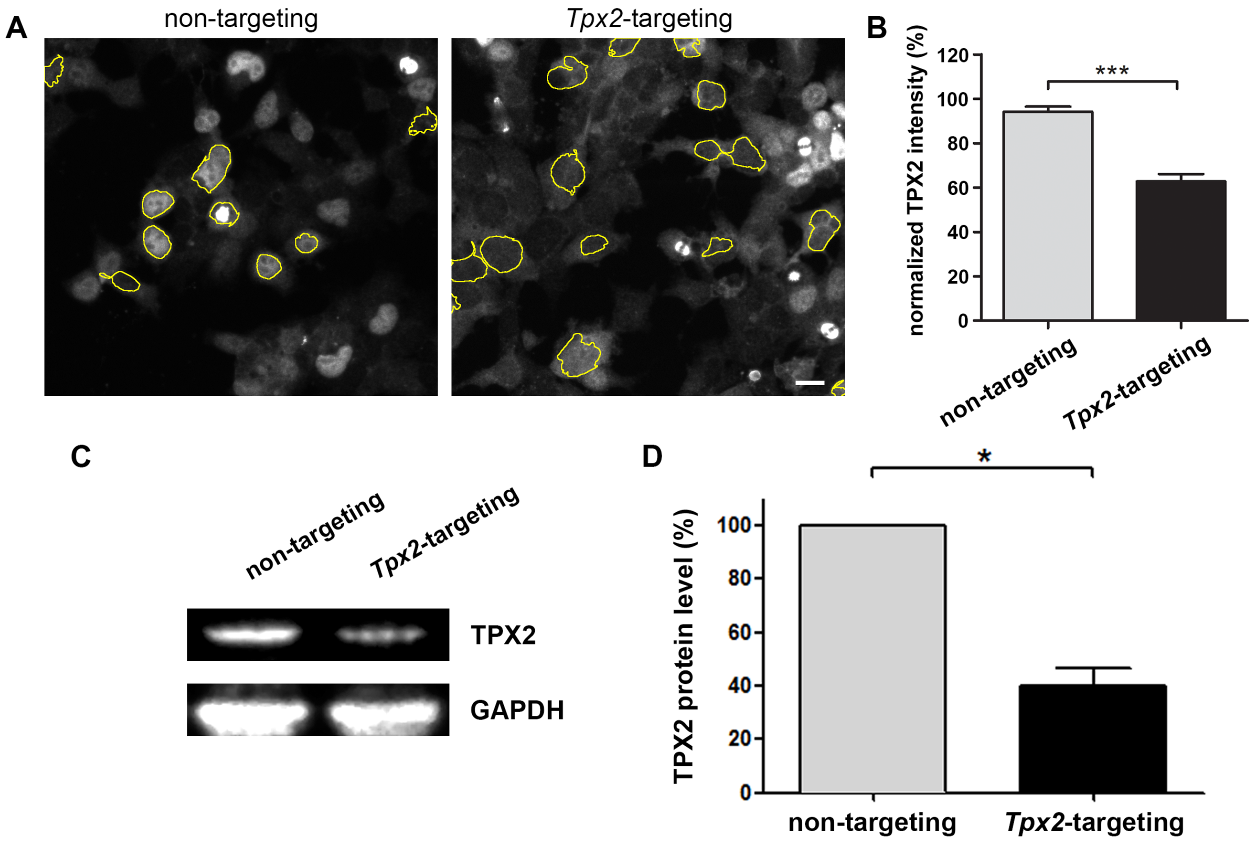

Figure S1. TPX2 knockdown validation. (A) Mouse P19 cells transfected with non-targeting or *Tpx2*-targeting shRNA plasmids for 2 days, fixed, and immunofluorescence stained with anti-TPX2 antibody. Yellow regions indicate the nuclei of transfected cells as determined by the DAPI and EGFP signal. Scale bar represents 10 µm. (B) Quantification of TPX2 protein level in interphase cells as shown in A. Only TPX2 signal inside the yellow regions was used for the quantification. TPX2 intensity was normalized to the mean TPX2 intensity in empty vector-transfected P19 cells. *** $p < 0.01$, two-tailed Student's t-test. Error bars represent SEM from 3 independent repeats. (C) Immunoblot using antibody against TPX2 with P19 cell lysates expressing non-targeting or *Tpx2*-targeting shRNA. Antibody against GAPDH was used as the loading control. (D) Quantification of TPX2 protein level. TPX2 protein level was corrected using GAPDH protein level and normalized to the non-targeting control. * $p < 0.05$, two-tailed Student's t-test. Error bars represent SEM from 3 independent repeats.

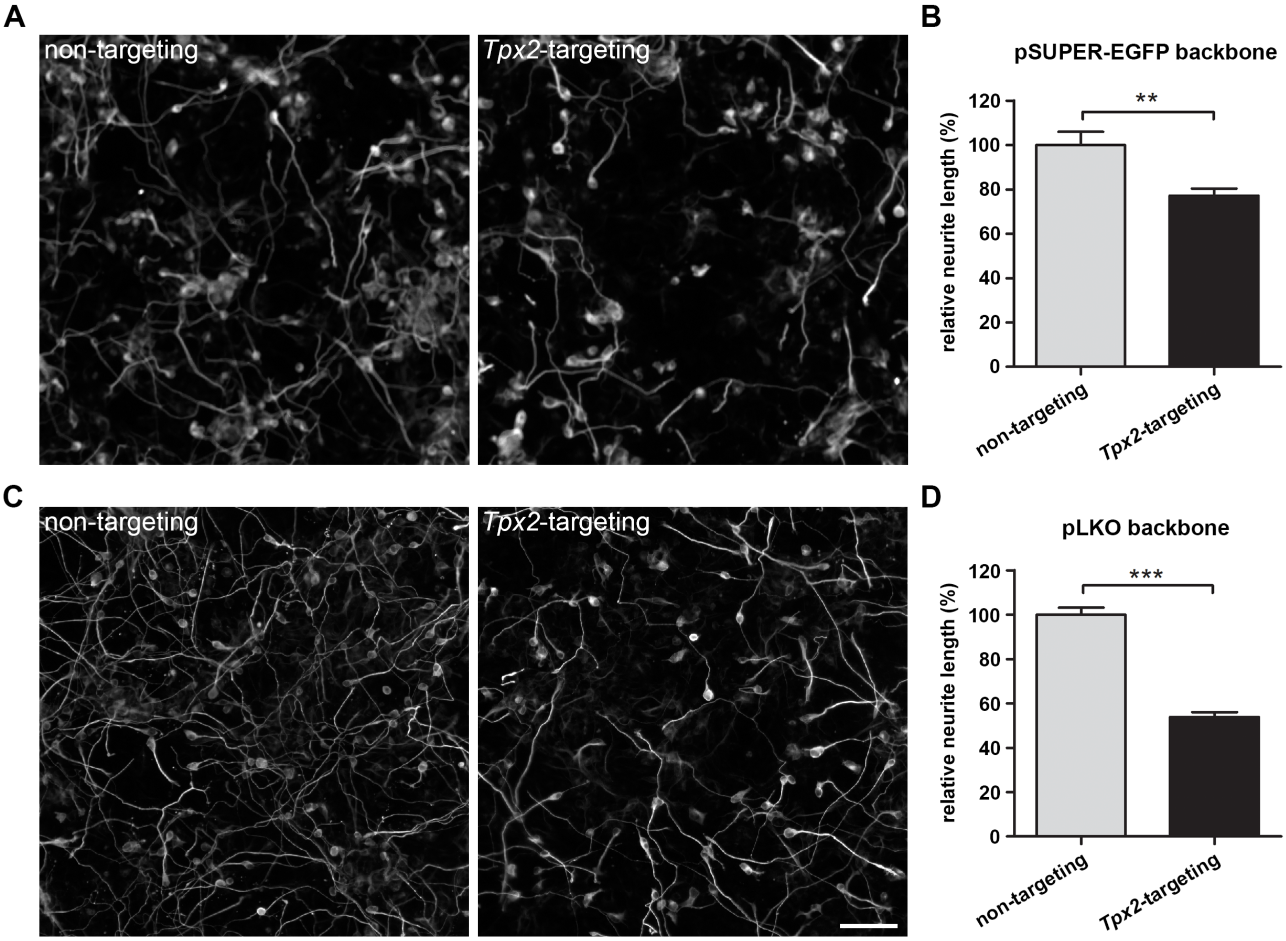

Figure S2. TPX2 depletion reduces neurite elongation in embryonal carcinoma cells differentiated neurons. (A) Images of P19 differentiated neurons expressing non-targeting (left) or *Tpx2*-targeting (right) shRNA from the pSUPER vector backbone. (B) Quantification of P19 total neurite length shown in A. (C) Images of P19 differentiated neurons expressing non-targeting (left) or *Tpx2*-targeting (right) shRNA from the pLKO vector backbone. (D) Quantification of P19 total neurite length shown in C. ** $p < 0.01$, *** $p < 0.001$, two-tailed Student's *t*-test. Error bars represent SEM from 3 independent repeats. All images have the same scale and the scale bar represents 100 μm.

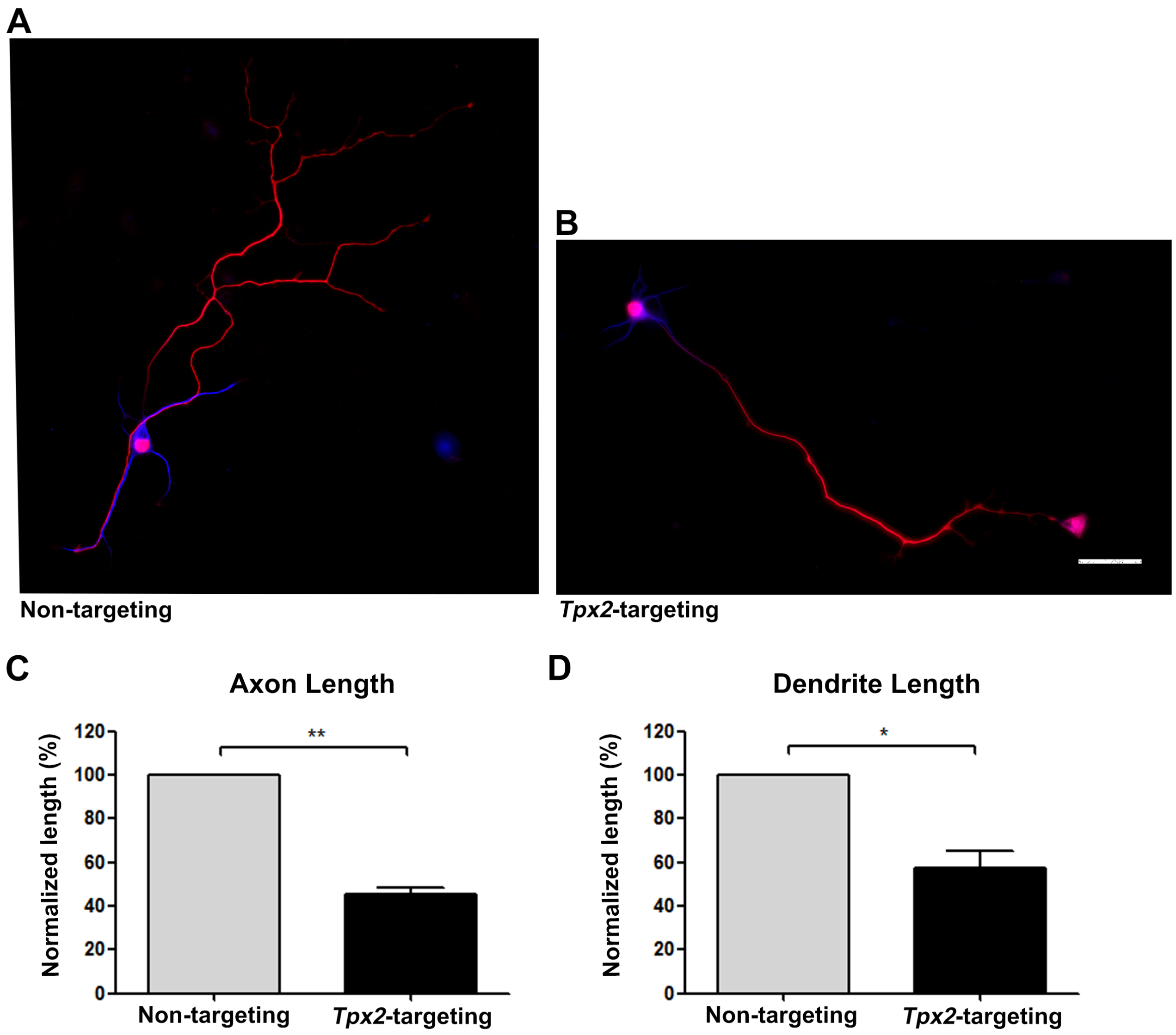

Figure S3. TPX2 depletion reduces both axon and dendrite length. Dissociated hippocampal neurons were transfected with plasmid expressing control non-targeting shRNA (A) or *Tpx2*-targeting shRNA (B) at 2DIV and fixed at 5DIV. Fixed neurons were immunofluorescence stained with axon-specific (SMI312, red) and dendrite-specific (MAP2, blue) antibody. The scale bar presents 100 μm. Quantification of average axon length (C) and average dendrite length (D) of control and TPX2-depleted neurons. *, $p < 0.05$, **, $p < 0.01$, by two-tailed Student's *t*-test. Error bars represent SEM from 3 independent experiments. More than 90 neurons were analyzed from 3 independent repeats for both the control and TPX2-depleted group.

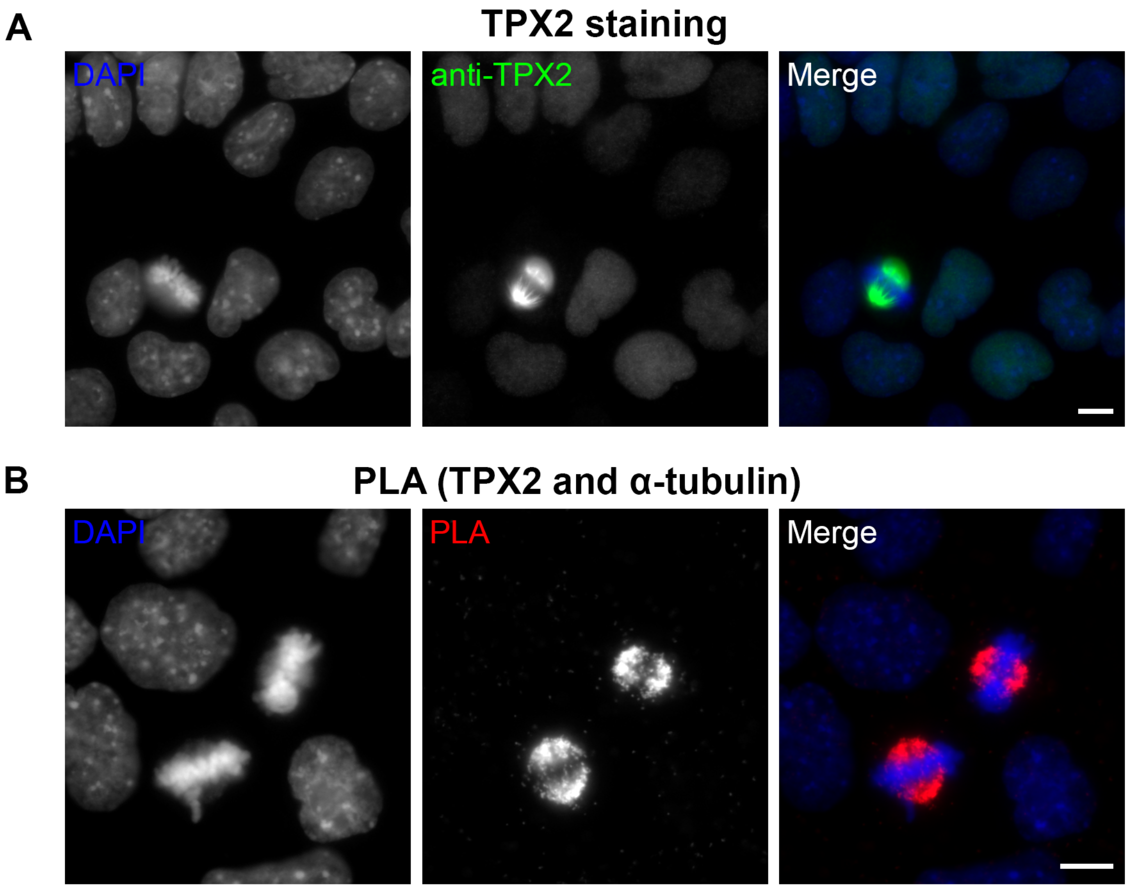

Figure S4. TPX2 binds to microtubules during mitosis but not during interphase.
(A) Images of mitotic P19 cells immunofluorescence stained with anti-TPX2 antibody and DAPI. The merged image is shown on the right. TPX2 localizes within the nucleus during interphase and localizes to the spindle MTs during mitosis. Our observation is consistent with previous reports and demonstrates the specificity of the TPX2 antibody in mouse cells. (B) Images of *in situ* proximity ligation assay (PLA) for TPX2 and α-tubulin in mitotic P19 cells. PLA signals were only present in cells during mitosis but not during interphase. All scale bars represent 10 μm.

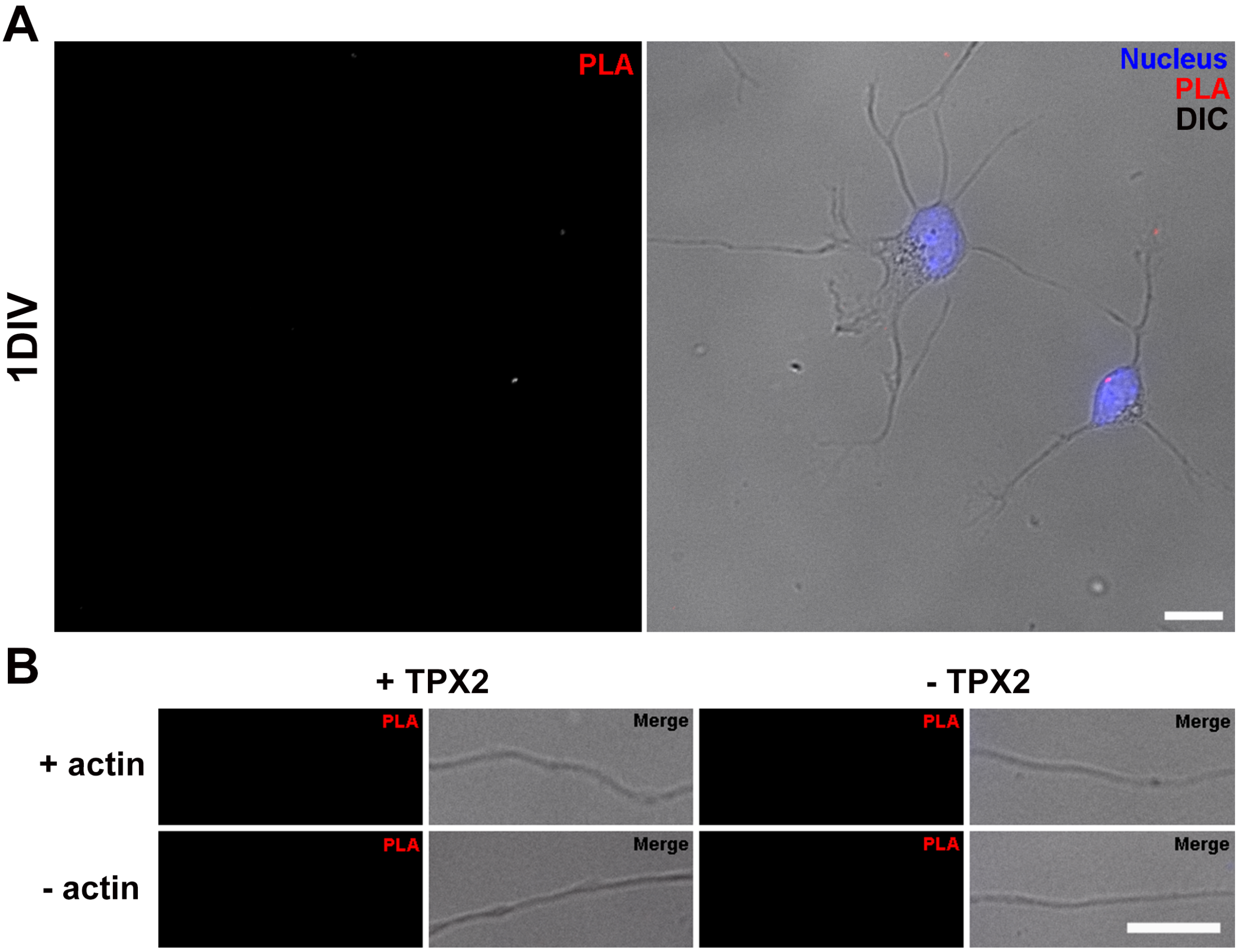

Figure S5. TPX2 does not interact with the actin cytoskeleton along the neurite shaft.
(A) Representative images of *in situ* proximity ligation assay (PLA) for TPX2 and actin in 1DIV dissociated hippocampal neurons. Nuclei were detected by DAPI and cell outline was revealed using differential interference contrast (DIC) microscopy. PLA puncta were never observed in the soma or along the neurite shaft. (B) Control experiments showing the specificity of PLA. All scale bars represent 10 µm.

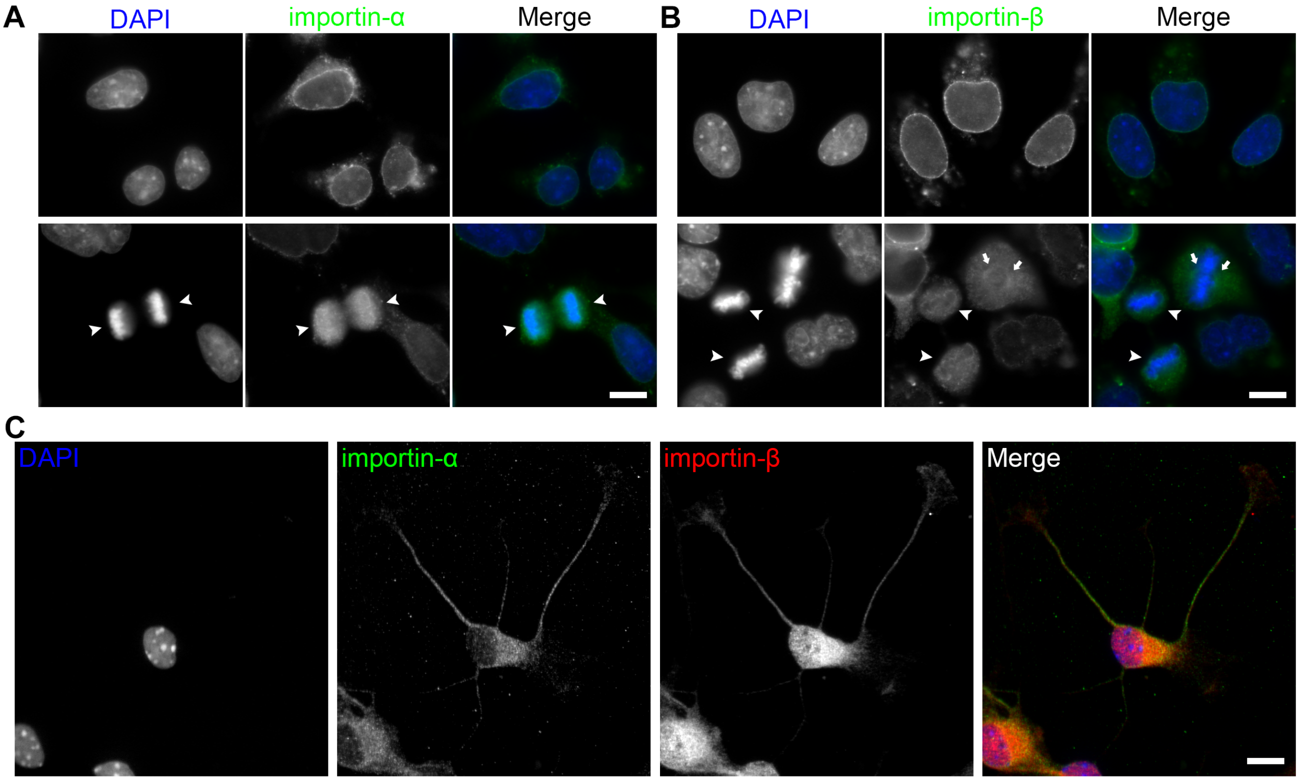

Figure S6. The localization of importin-α and β in mitotic cells and hippocampal neurons. Mitotic P19 cells were pre-extracted with 0.1% triton X-100 in PIPES buffer and fixed in 3.7% formaldehyde for immunofluorescence staining. Importin-α (A) and importin-β (B) both localized to the nuclear envelope during interphase (top row) and around chromosomes during anaphase (bottom row, arrowheads). Importin-β was also observed at spindle poles (arrow). Our observation is consistent with previous reports and demonstrates the specificity of these importin antibodies. (C) 1DIV hippocampal neurons were directly fixed in 3.7% formaldehyde for immunofluorescence staining. Both importin-α and β were detected in the soma and along the neurite. DAPI stain was used to detect the nucleus. All scale bars represent 10 μm.

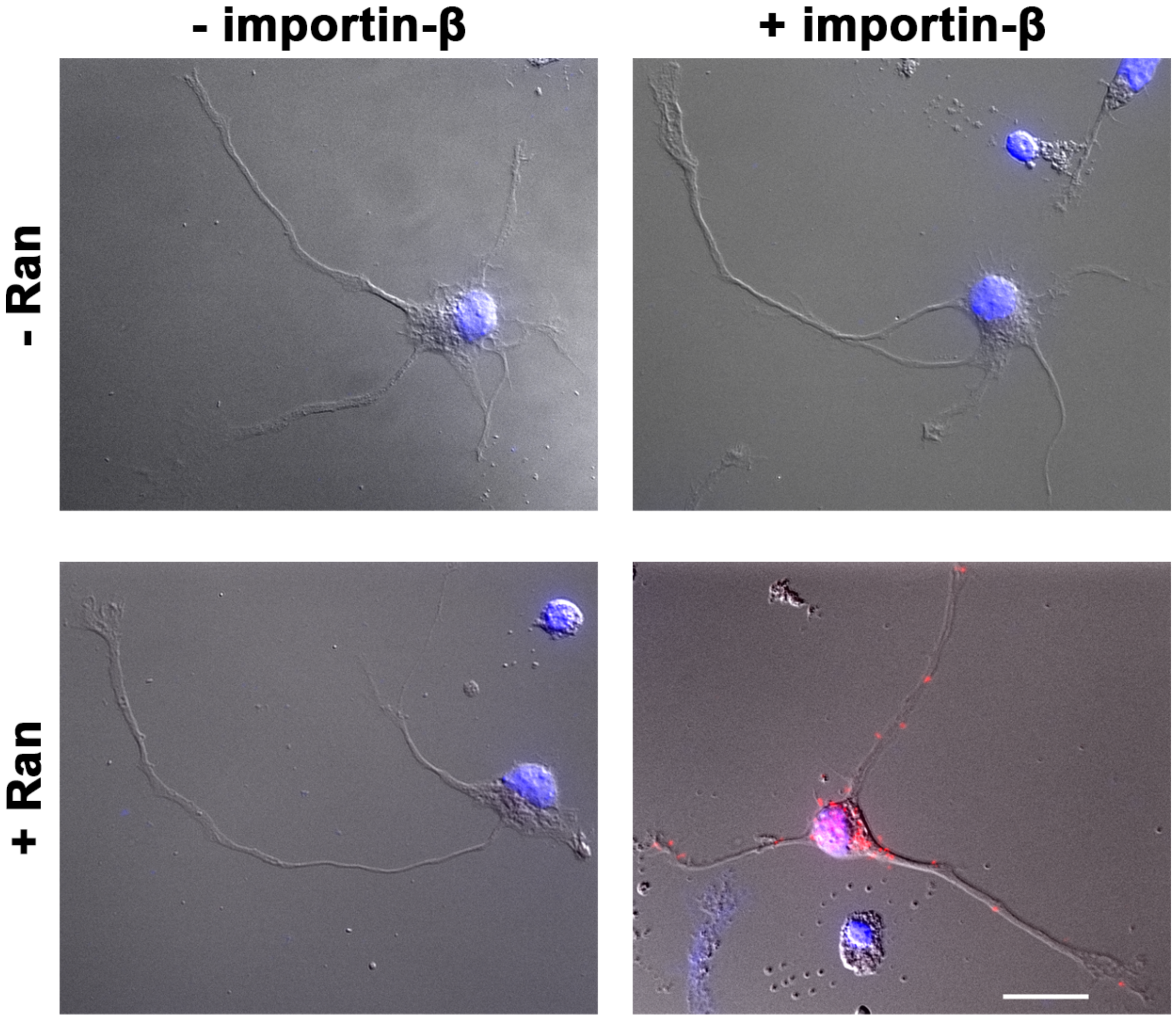

Figure S7. Ran-importin-β complexes can be detected in the cytoplasm of hippocampal neurons. Representative images of *in situ* proximity ligation assay (PLA) for Ran and importin-β in 2DIV dissociated hippocampal neurons. Nuclei were detected by DAPI and cell outline was revealed using differential interference contrast (DIC) microscopy. PLA puncta were present at neurite tip, along the neurite shaft, and in the soma only when anti-Ran (which recognizes both RanGTP and RanGDP) and anti-importin-β antibodies were both present. All images have the same scale and the scale bar represents 20 μm.

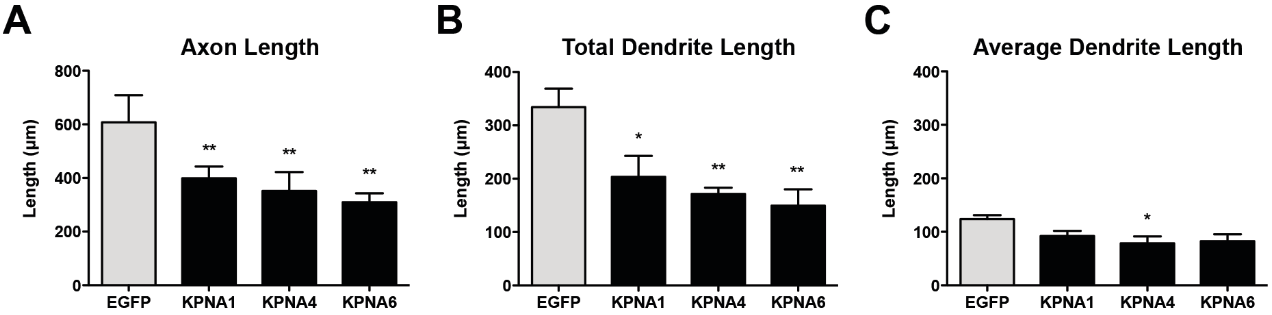

Figure S8. Overexpressing importin-α reduces axon and dendrite length. Dissociated hippocampal neurons transfected with plasmid overexpressing KPNA1, KPNA4, or KPNA6 at 2DIV and fixed at 4DIV. Quantification of average axon length (A), total dendrite length (B), and average dendrite length (C). *, $p < 0.05$, ** $p < 0.01$, one-way ANOVA followed by Dunnett's comparison to the control group. Error bars represent SEM from 3 independent experiments.

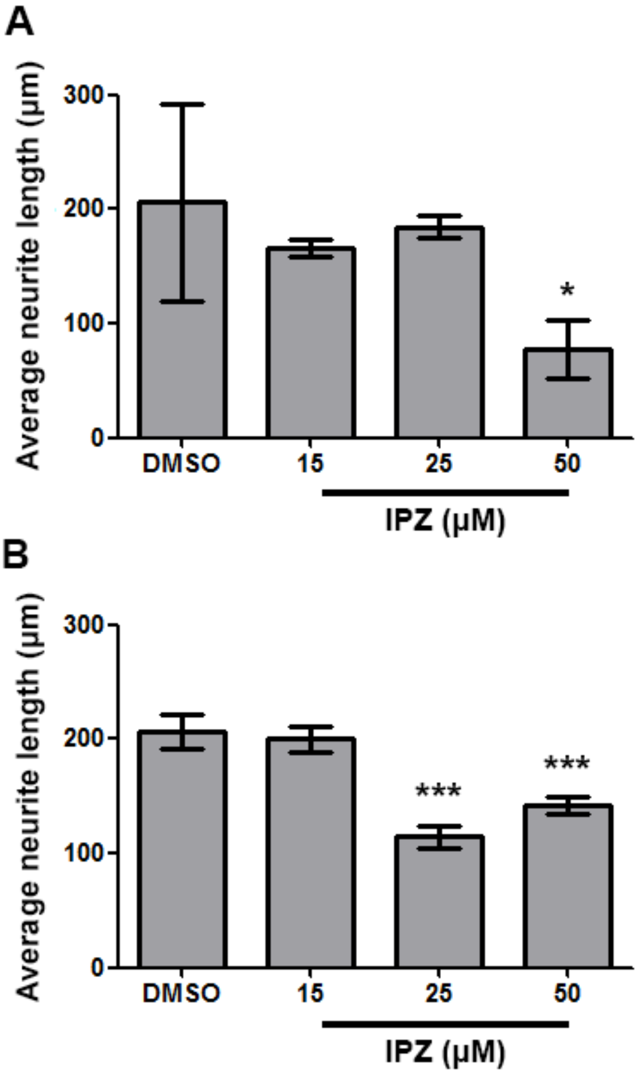

Figure S9. Importazole induces neurite retraction. 2DIV dissociated hippocampal neurons were treated with 15, 25, 50 µM of importazole (IPZ) for 1 (A) or 2 hours (B) before fixation. Fixed neurons were immunofluorescence stained with antibody against β-III-tubulin for neurite length quantification. * $p < 0.05$, *** $p < 0.001$, one-way ANOVA followed by Dunnett's comparison to the DMSO control group. Error bars represent SEM from 3 independent repeats.

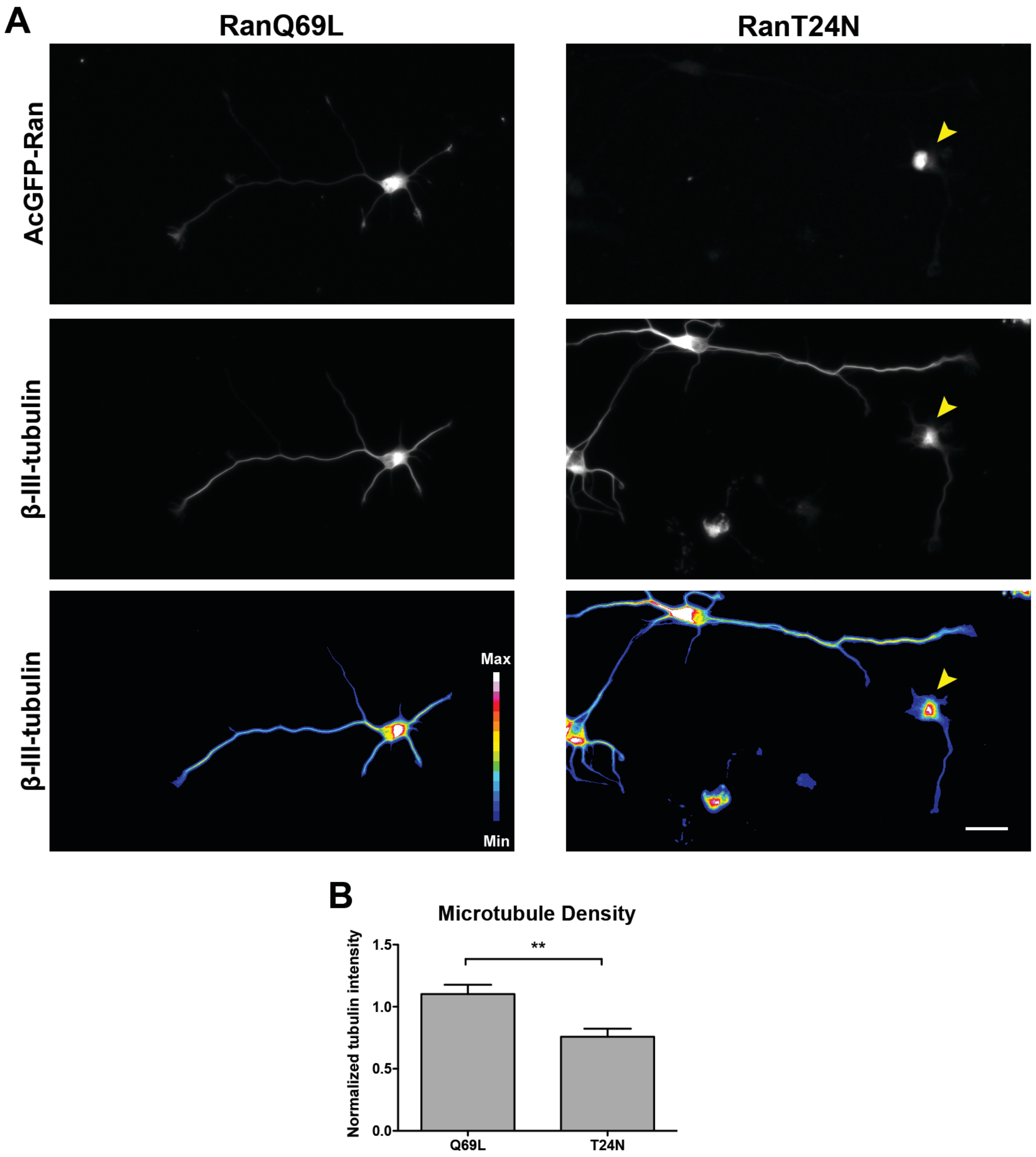

Figure S10. Ran affects microtubule density along the neurite. (A) Dissociated hippocampal neurons were transfected with plasmid overexpressing RanQ69L (left column) or RanT24N (right column) before plating and fixed at 2DIV. The AcGFP signal (top), β-III-tubulin signal (middle), and pseudo-colored β-III-tubulin signal (bottom) are shown. The yellow arrows denote a transfected neuron. Notice that untransfected neurons have a higher β-III-tubulin signal along the neurite than that of a RanT24N-transfected neuron. The scale bar presents 50 μm. (B) Quantification of microtubule density along the primary neurite using the β-III-tubulin signal. The signal of β-III-tubulin along the primary neurite of transfected neurons was normalized against that of the untransfected neurons. ** $p < 0.01$, two-tailed Student's t-test. Error bars represent SEM.
